# Effect of Argon-Based Atmospheric Pressure Plasma Treatment on Hard Tissue Formation on Titanium Surface

**DOI:** 10.3390/ijms22147617

**Published:** 2021-07-16

**Authors:** Satoshi Komasa, Tetsuji Kusumoto, Rina Hayashi, Seiji Takao, Min Li, Sifan Yan, Yuhao Zeng, Yuanyuan Yang, Hui Hu, Yasuyuki Kobayashi, Akinori Agariguchi, Hisataka Nishida, Yoshiya Hashimoto, Joji Okazaki

**Affiliations:** 1 Department of Removable Prosthodontics and Occlusion, Osaka Dental University, 8-1 Kuzuha-hanazono-cho, Hirakata, Osaka 573-1121, Japan; hayashi-r@cc.osaka-dent.ac.jp (R.H.); takao-s@cc.osaka-dent.ac.jp (S.T.); liminmin0529@gmai.com (M.L.); yan-z@cc.osaka-dent.ac.jp (S.Y.); zeng-y@cc.osaka-dent.ac.jp (Y.Z.); yang-y@cc.osaka-dent.ac.jp (Y.Y.); huhui0726@outlook.com (H.H.); akinori@agariguchi.com (A.A.); joji@cc.osaka-dent.ac.jp (J.O.); 2 Department of Japan Faculty of Health Sciences, Osaka Dental University, 1-4-4, Makino-honmachi, Hirakata-shi, Osaka 573-1121, Japan; kusumoto@cc.osaka-dent.ac.jp; 3 Osaka Research Institute of Industrial Science and Technology, Morinomiya Center, 1-6-50, Morinomiya, Joto-ku, Osaka 536-8553, Japan; kobaya@omtri.or.jp; 4 Department of Advanced Hard Materials, The Institute of Scientific and Industrial Research (ISIR), Osaka University, 8-1 Mihogaoka, Ibaraki, Osaka 567-0047, Japan; hnishida@sanken.osaka-u.ac.jp; 5 Department of Biomaterials, Osaka Dental University, 8-1 Kuzuha-hanazono-cho, Hirakata, Osaka 573-1121, Japan; yoshiya@cc.osaka-dent.ac.jp

**Keywords:** argon plasma treatment, titanium implant, reactive oxygen species, hard tissue differentiation, rat bone marrow, bone formation

## Abstract

In this paper, we suggest that the atmospheric pressure plasma treatment of pure titanium metal may be useful for improving the ability of rat bone marrow cells (RBMCs) to induce hard tissue differentiation. Previous studies have reported that the use of argon gas induces a higher degree of hard tissue formation. Therefore, this study compares the effects of plasma treatment with argon gas on the initial adhesion ability and hard tissue differentiation-inducing ability of RBMCs. A commercially available titanium metal plate was used as the experimental material. A plate polished using water-resistant abrasive paper #1500 was used as the control, and a plate irradiated with argon mixed with atmospheric pressure plasma was used as the experimental plate. No structural change was observed on the surface of the titanium metal plate in the scanning electron microscopy results, and no change in the surface roughness was observed via scanning probe microscopy. X-ray photoelectron spectroscopy showed a decrease in the carbon peak and the formation of hydroxide in the experimental group. In the distilled water drop test, a significant decrease in the contact angle was observed for the experimental group, and the results indicated superhydrophilicity. Furthermore, the bovine serum albumin adsorption, initial adhesion of RBMCs, alkaline phosphatase activity, calcium deposition, and genetic marker expression of rat bone marrow cells were higher in the experimental group than those in the control group at all time points. Rat distal femur model are used as in vivo model. Additionally, microcomputed tomography analysis showed significantly higher results for the experimental group, indicating a large amount of the formed hard tissue. Histopathological evaluation also confirmed the presence of a prominent newly formed bone seen in the images of the experimental group. These results indicate that the atmospheric pressure plasma treatment with argon gas imparts superhydrophilicity, without changing the properties of the pure titanium plate surface. It was also clarified that it affects the initial adhesion of bone marrow cells and the induction of hard tissue differentiation.

## 1. Introduction

Currently, research and development is underway to improve the achievement of osseointegration of implants with the aim of expanding the range of the application of biocompatible materials to bone tissues [1,2,3]. Vandrocova et al. reported that surface-controlled dental materials have a significant effect on the initial cell adhesion and bone differentiation [4]. Undifferentiated mesenchymal cells present in the tissue surrounding an implant are known as scaffold-dependent cells. It was reported that the osseointegration of implants depends on the surface properties, such as the surface morphology and chemical composition of the various materials that serve as scaffolds for undifferentiated mesenchymal cells [5,6,7,8,9,10]. Titanium, the main material used for implants, is known as a biomaterial, and it was clarified that it does not directly bind to the implant cavity; however, it is bioactive owing to the modification of the surface structure. It was reported that it may regulate bone formation at the bone implant body interface [11,12].

In recent years, it was emphasized that the surface wettability affects the dynamics of osteoblasts and, by extension, osseointegration; furthermore, implants exhibiting hydrophilicity are already available in the market. Many in vitro and in vivo studies have been conducted on the relationship between hydrophilicity and cell adhesion [13,14,15,16,17,18]. In the past, we have conducted many studies on the correlation between the wettability of the material surface and various cells [19,20,21,22,23,24,25,26,27]. It was clarified that, on treating the titanium surface, titanium alloy, and zirconia material with a concentrated alkali, the shape of the material surface changed, and the contact angle of the material surface, was reduced [19,28,29,30]. Additionally, it was clarified (based on in vitro and in vivo studies) that this structure contributes to the initial adhesion of RBMCs and the improvement in the ability to induce hard tissue differentiation [31]. Generally, the surface shape is considered to play an important role in cell differentiation and expression morphology; however, it is considered that the changes in the surface properties are crucial for the initial adhesion of proteins involved in cellular processes and osseointegration [13,14,15]. As a surface chemical modification method for imparting hydrophilicity to the surface of a material, an atmospheric pressure plasma treatment method, an ultraviolet irradiation method, and a hydrogen peroxide solution immersion method have been proposed. The atmospheric pressure plasma method is a method of creating an ionized state at a low temperature; because it is possible to control the surface energy and the surface of surface home appliances by introducing a functional group on the surface. Therefore, the scope of application of the atmospheric pressure plasma method to biomaterials is expanding [32,33]. It was reported that the UV irradiation method imparts hydrophilicity to the titanium surface owing to the photocatalytic effect of titanium oxide and is effective for the proliferation and differentiation of osteoblasts [34,35,36].

In our previous studies, we have reported on the UV treatment and atmospheric pressure plasma treatment of titanium surfaces [23,37,38]. It was clarified that by applying UV treatment and atmospheric pressure plasma treatment to a titanium surface, the carbon content on the material surface could be reduced, and hydrophilicity was imparted to the surface without changing the surface structure. It was also shown that the surface of this material induced protein adsorption, early adhesion, and differentiation induction of bone marrow cells, as well as new bone formation in the tissues surrounding the implant. Furthermore, in the evaluation using the QCM system, as a result of each treatment of the pure titanium metal surface, it was reported that the adhesion rate of bone marrow cells and proteins was higher in the atmospheric pressure plasma treatment group than that in the UV treatment group [39].

The data show that the reactive oxygen species (ROS) on the material surface are less in the plasma treatment group in the background, and it is considered that a desirable environment for cell growth is established. ROS activate osteoclast differentiation and osteocyte apoptosis (+), and inhibit osteoblast activity (−) inducing bone resorption; antioxidants activate osteoblast differentiation (+) and inhibit osteoclast activity and osteocyte apoptosis (−) inducing bone formation. We have also succeeded in the UV treatment and plasma treatment of titanium surfaces with nanostructures, and the changes in the surface structure of nanostructures and in the surface texture induced by each treatment are interdependent, resulting in high hardness [23]. It was reported that it could induce tissue differentiation and antibacterial properties. Additionally, we succeeded in imparting hydrophilicity to the material surface by conducting UV treatment and plasma treatment on NANOZR, which is attracting much attention as an implant material for metal-allergic patients, and clinicians use an important method to impart hydrophilicity [40,41]. However, it is difficult to maintain the hydrophilic state of the material surface, and further improvements are thus required.

The low-temperature atmospheric pressure plasma treatment that creates an ionized state at a low temperature is a method that can control the surface properties, such as surface energy and surface load by modifying a functional group on the surface in addition to the cleaning effect on the material surface [42,43]. For this reason, its scope of application to biomaterials is expanding. Among these, plasma treatment performed in the presence of argon is also called decompression argon plasma treatment, and it can possibly be used widely as a surface treatment method for implant materials. It was reported that pure titanium metal is subjected to plasma treatment in an environment mediated by oxygen or argon to further enhance the hydrophilicity and improve the initial adhesiveness and differentiation characteristics of various cells [44,45,46,47]. The plasma device used in this study is a piezo brush. We have already conducted a study regarding the surface of hydrophilically treated materials and the biocompatibility attained using this device; thus, the clinical usefulness of this device was successfully demonstrated [40,48]. Most of the plasma devices consist of large machines [49,50], and there are many inconveniences assuming that they are to be used by clinical dentists. In contrast, the plasma apparatus used in the present study is comparatively small and easy to use. The piezo brush is a device that changes the nozzle of the device and enables plasma processing in various gaseous environments. That is, it is possible to treat a titanium surface by decompression argon plasma treatment, and this is expected to create a new material with the aim of further improving biocompatibility. The piezo brush used in this experiment is a handy type plasma device, which is useful as a device used by clinical dentists. This time, a multi-gas nozzle can be attached to this plasma device, and it is possible to use gas together. The use of argon gas significantly increases the premature formation of peri-implant bone, which is expected to make this device even more useful to clinicians.

The aim of the present study is to investigate the mechanism by which piezo brush plasma treatment with argon gas on a titanium surface affects its initial adhesion and ability to induce hard-tissue differentiation in RBMCs, in vitro and in vivo. It is expected that the results of this experiment will be useful as a prosthetic treatment option and will help the patients suffering from various disorders.

## 2. Results

### 2.1. Surface Characterization

**Figure 1:**
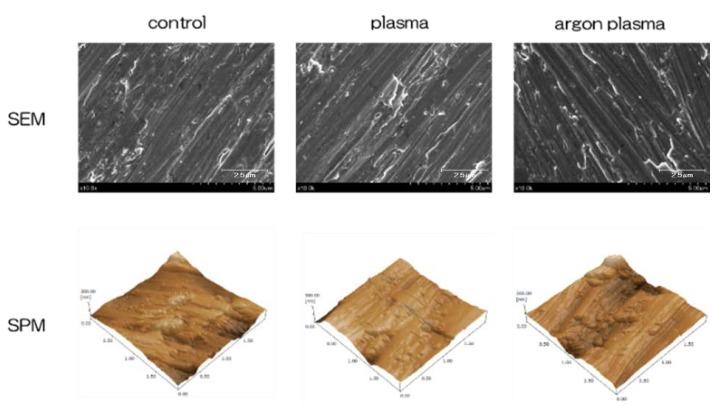
Scanning electron microscopy (SEM) and scanning probe microscopy (SPM) observations show no structural changes on the titanium surface after plasma and argon plasma treatment.

**Figure 2:**
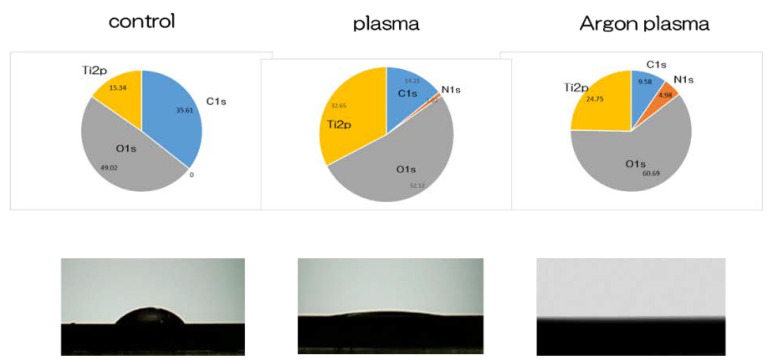
Based on the results of the X-ray photoelectron spectroscopy (XPS) analysis, an increase in the O_2_ peak on the titanium surface was observed due to plasma treatment. In contrast, the C1s peak height on the titanium surface was decreased due to the plasma treatment. The C1s peak showed the lowest value in the argon plasma-modified group. It was clarified that the contact angle of the material surface was reduced due to plasma treatment; this was the lowest value in the argon plasma-modified group. The untreated group showed 62°, the plasma-treated group showed 8°, the argon gas group showed 0°, and the argon gas group showed the lowest value.

Figure 1 shows the surface analysis results of titanium surface. Scanning electron microscope (SEM) evaluation showed no structural changes on the material surface due to argon-based plasma and plasma treatment (Figure 1). In addition, scanning probe microscopy (SPM) analysis did not show any change in the surface roughness of the test disk (Figure 1). Based on the results of X-ray photoelectron spectroscopy (XPS) analysis, the surface treatment increased the peaks of O1s and N1s. It showed the highest value in the argon plasma treatment group. In contrast, the C1s peak height on the titanium surface was reduced by plasma treatment (Figure 2). The C1s peak showed the lowest value in the argon plasma treatment group. It was clarified that the contact angle of the material surface was reduced by the plasma treatment. The untreated group showed 62°, the plasma-treated group showed 8°, the argon gas group showed 0°, and the argon gas group showed the lowest value (Figure 2).

### 2.2. Evaluation of Protein Adsorption on the Titanium Surface

**Figure 3:**
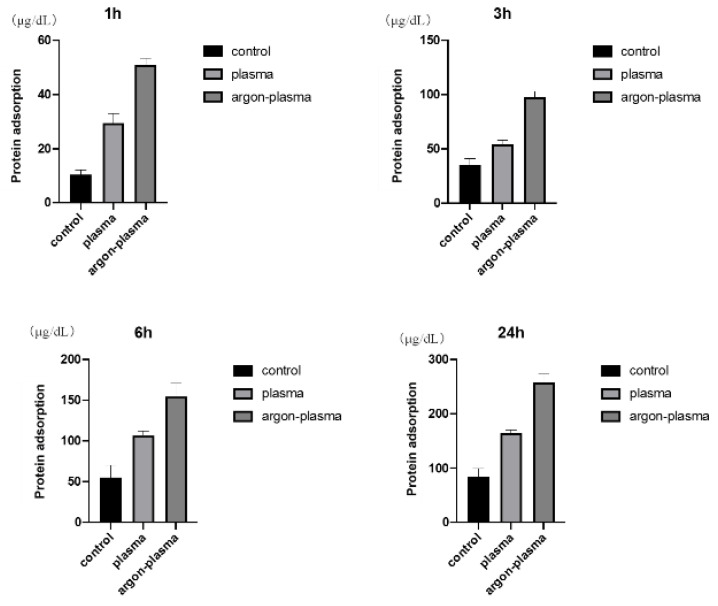
The results of the adhesion examination of BSA among the three groups are shown. The BSA adsorption was significantly higher in the test group than in the control group. In addition, the highest value was observed in the group modified with argon plasma.

The results of the adhesion examination of BSA among the three groups are shown (Figure 3). The BSA adsorption was significantly higher in the test group than in the control group. In addition, the highest value was observed in the group modified with argon plasma.

### 2.3. Effects of the Titanium Surface on Cell Adhesion and Morphology in RBMCs

**Figure 4:**
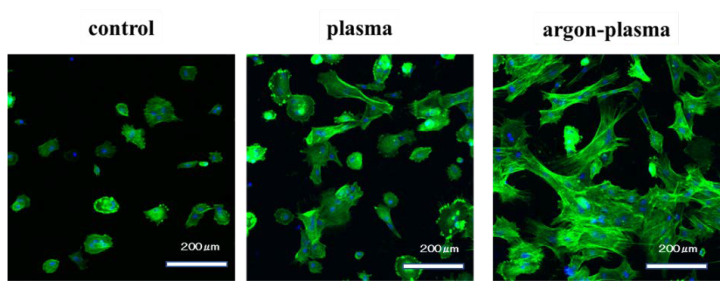
The morphology of RBMCs on the titanium surface after 6 h of culture was observed using a fluorescence microscope. It was confirmed that the RBMCs adhered to the surface of the materials for all three groups.

**Figure 5:**
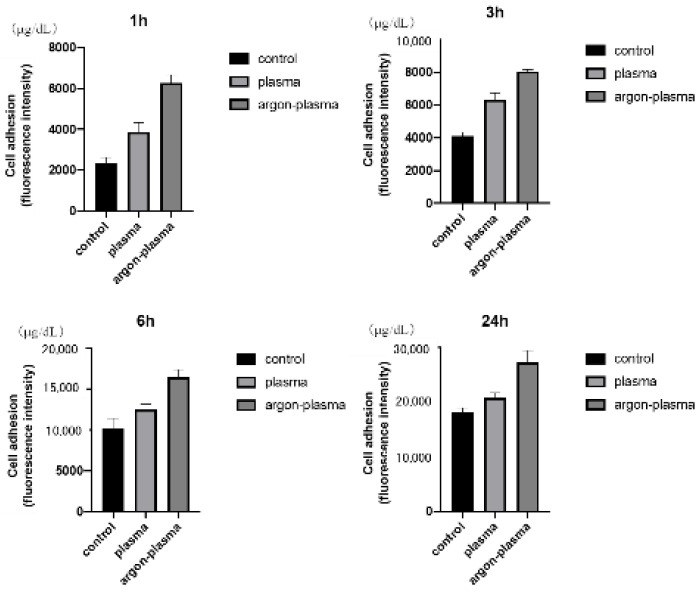
After argon-plasma treatment on the material surface, the number of adhered RBMCs was statistically significantly higher than that in the control group. In addition, the highest value was observed in the argon plasma treatment group, among all three groups. At all measurement time points, the adhesion number of RBMCs in the argon plasma-treated group was significantly the highest among all three groups.

The morphology of RBMCs on the titanium surface after 6 h of culture was observed using a fluorescence microscope. It was confirmed that the RBMCs adhered to the surface of the materials for all three groups (Figure 4). After argon-plasma treatment on the material surface, the number of adhered RBMCs was statistically significantly higher than that in the control group. In addition, the highest value was observed in the argon plasma treatment group, among all three groups. At all measurement time points, the adhesion number of RBMCs in the argon plasma-treated group was the highest, significantly, among all three groups (Figure 5).

### 2.4. Argon Plasma Treatment-Induced Bone Differentiation on the Titanium Surface In Vitro

**Figure 6:**
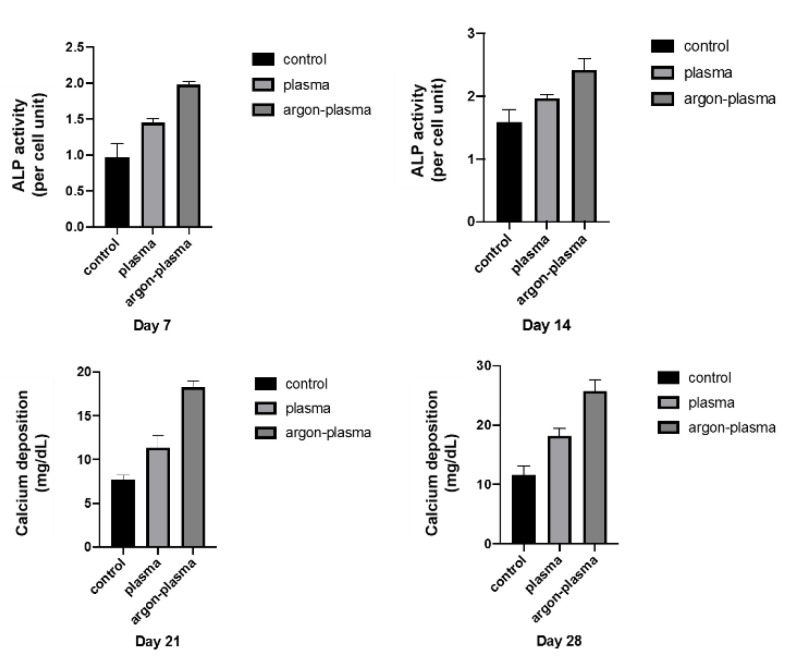
Alkaline phosphatase (ALP) expression in bone marrow cells at days 7 and 14, after the start of culture, was significantly higher on the material surface of the argon plasma-treated group, among all three groups. The amount of calcium deposited on the material surface at days 21 and 28 after the culture incubation, was significantly the highest for the argon plasma-treated group, among all three groups.

**Figure 7:**
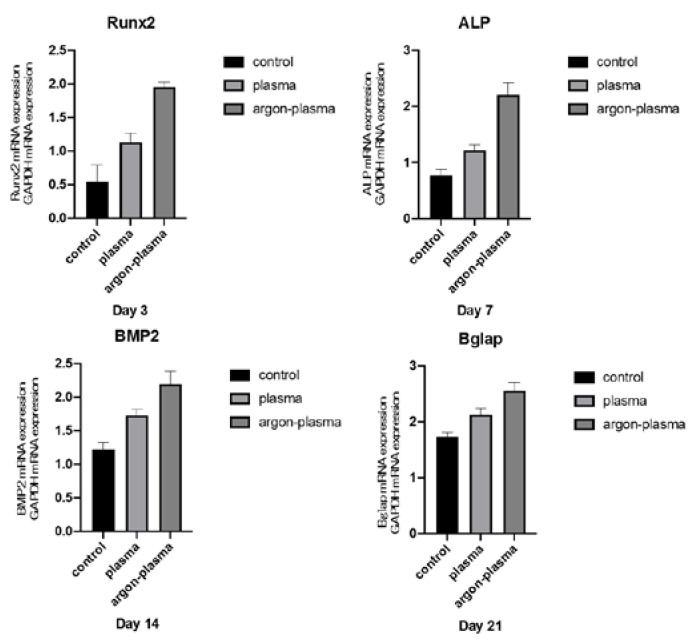
The gene expression related to the induction of hard tissue differentiation on the material surface of the samples in the test and control groups were analyzed. In this experiment, the assay was performed at a measurement time specific to each gene. Significantly higher gene expression was observed on the material surface of the samples in the argon plasma-treated group, among all three groups, at all measurement time points.

Alkaline phosphatase (ALP) expression in bone marrow cells at days 7 and 14, after the start of culture, was significantly higher on the material surface of the argon plasma-treated group, among all three groups (Figure 4). The amount of calcium deposited on the material surface at days 21 and 28 after the culture incubation, was significantly the highest for the argon plasma-treated group, among all three groups (Figure 6). The gene expression related to the induction of hard tissue differentiation and angiogenesis on the material surface of the samples in the test and control groups was analyzed. In this experiment, the assay was performed at a measurement time specific to each gene. Significantly higher gene expression was observed on the material surface of the samples in the argon plasma-treated group, among all three groups, at all measurement time points (Figure 7). 

### 2.5. Cell Intracellular ROS Level of RBMCs on Titanium Surface

**Figure 8:**
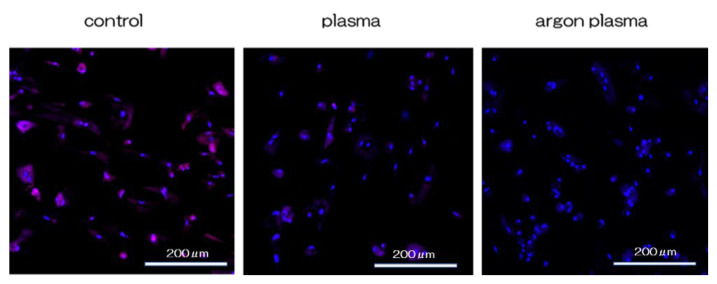
RBMCs on the titanium surface showed significantly higher levels of intra cellular ROS, than the RBMCs on the plasma-treated titanium surface, and the lowest ROS levels in the argon plasma-treated group. These results indicate that argon plasma treatment may enhance the antioxidant properties of titanium surfaces.

RBMCs on the titanium surface showed significantly higher levels of intracellular ROS, than the RBMCs on the plasma-treated titanium surface, and the lowest ROS levels in the argon plasma-treated group. These results indicate that argon plasma treatment may enhance the antioxidant properties of titanium surfaces (Figure 8). The RBMCs on the titanium surface showed significantly higher levels of intracellular ROS than the RBMCs on the plasma-treated titanium surface, and the lowest ROS levels in the argon plasma-treated group.

### 2.6. Evaluation of the Amount of New Bone Formation in the Tissue Surrounding the Titanium Implant Placement In Vivo

**Figure 9:**
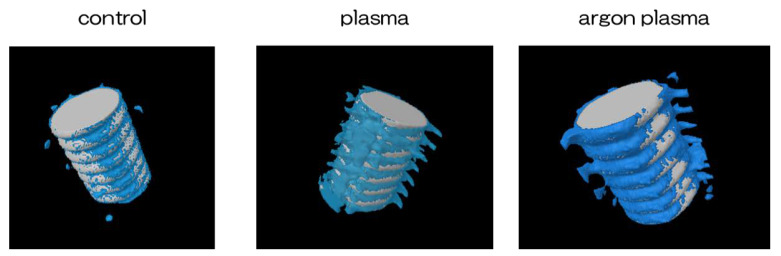
Trabecular microarchitecture was more prominent in the area of the material surface for the test group, than for the control group. White; implant body, blue; bone.

**Figure 10:**
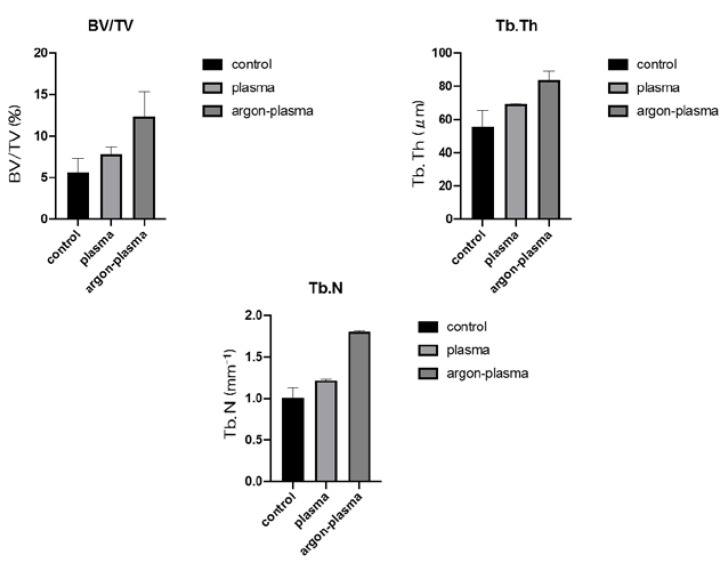
Furthermore, the ratio of bone mass to total mass (BV/TV), average trabecular number (Tb.N), and average trabecular thickness (Tb.Th), were the highest, significantly, for the material surface of the argon plasma-treated group, among all three groups. The implants promoted osteogenic activity (*p* < 0.05).

Trabecular microarchitecture was more prominent in the area of the material surface for the test group, than for the control group (Figure 9). Furthermore, the ratio of bone mass to total mass (BV/TV), average trabecular number (Tb.N), and average trabecular thickness (Tb.Th), were significantly the highest for the material surface of the argon plasma-treated group, among all three groups. The implants promoted osteogenic activity (*p* < 0.05) (Figure 10).

**Figure 11:**
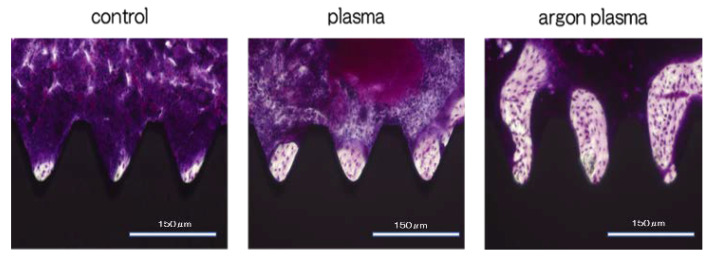
Furthermore, the amount of new bone formation was confirmed based on the longitudinal sections. Greater amount of newly formed bone was observed around the implants in the argon plasma-treated group, among all three groups. Black; implant body, purple; bone area.

**Figure 12:**
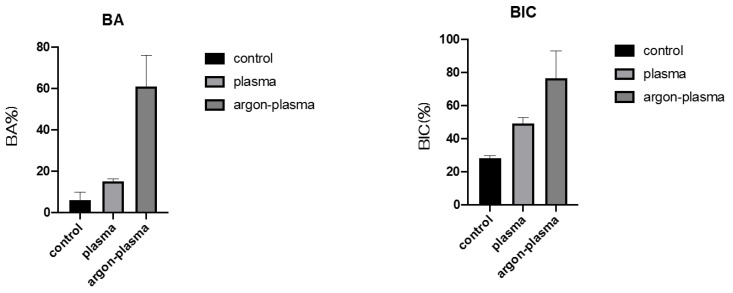
Quantitatively, histomorphometric analysis showed that the bone area ratio (BA) and bone-to-implant contact (BIC) on the material surface were significantly the highest for the argon plasma-treated group, among all three groups.

Furthermore, the amount of new bone formation was confirmed based on the longitudinal sections. As shown in Figure 11, a greater amount of newly formed bone was observed around the implants in the argon plasma-treated group, among all three groups. Quantitatively, histomorphometric analysis showed that the bone area ratio (BA) and bone-to-implant contact (BIC) on the material surface were significantly the highest for the argon plasma-treated group, among all three groups (Figure 12).

## 3. Discussion

The purpose of this study is to investigate the mechanism by which atmospheric pressure plasma treatment in an argon environment affects the bone formation in the tissue surrounding the implant, both in vitro and in vivo. It was clarified that through the atmospheric-pressure plasma treatment, the stain on the surface of the material was removed and hydrophilicity was imparted. Further, the oxygen and nitrogen contents of the material surface increased, showing the highest value in the argon plasma group. In addition, ROS evaluation revealed that the experimental conditions for the argon plasma-treated group were the most appropriate conditions for facilitating bone marrow cell growth. Correlating with these surface analysis results, the initial adhesion and hard tissue differentiation-inducing ability of proteins and bone marrow cells showed high values due to atmospheric pressure plasma treatment, and the highest values were observed for the argon plasma treatment group. In addition, the same treatment was performed on titanium screws that were implanted in the rat femur, and the in vivo analysis revealed the formation of the highest new bone in the argon plasma treatment group.

In recent years, implant surfaces have been clinically used by cleaning and sterilizing material surfaces with plasma equipped with UV equipment and high-temperature plasma sources. Some studies have shown beneficial results of modifying titanium surfaces with cold plasmas using argon gas [44,45,46,47]. By treating titanium surfaces with an argon plasma jet, some researchers have elongated the cell processes of bone marrow cells on the material surface and formed a new bone around the implant body embedded in the mandible of the beagle [48,51]. This amount was large. In a low-temperature plasma environment, a certain proportion of a mixture of argon and oxygen can generate large amounts of high-energy species such as reactive oxygen species (ROS) through energy transfer reactions, producing a high surface energy in the modified material [52]. It is necessary to examine the type of change generated on the material surface through the argon plasma treatment. SEM and previous atomic force microscopic evaluations have shown that the roughness of the implant surface in this study is similar to that of the other commercial products. The use of argon plasma treatment proved its effectiveness in cleaning contaminated pure titanium metal surfaces without altering the prevailing form of titanium. This is consistent with the results of a few previous studies [44,45,46,47]. As shown in the results, there was a significant change in the surface hydrophilicity and chemistry of the titanium surface. The wettability of the titanium surface was improved by applying atmospheric pressure plasma treatment, and the contact angle was the lowest in the argon plasma treatment group. The plasma generated using argon as the input gas, was shown to create superhydrophilic surfaces [44,45,46,47]. Based on the XPS analysis of the chemical functional groups, the amount of carbon decreased; however, the content of oxygen-binding compounds and nitrogen increased after argon plasma treatment. It was shown to be active enough to cleave C-H bonds in the surface layer to form radicals and generate various ROS on the surface. Carbon is usually identified on the surface as a result of the inevitable adhesion of the carbon-containing atmospheric components onto the surface. However, the adsorption of carbon on the surface can reduce biological activity and must be reduced to improve its activity. The presence of oxygen and nitrogen on the surface of the material was reported to be associated with the induction of bone marrow cell differentiation. It is inferred that the superhydrophilicity of the material surface and the presence of oxygen and nitrogen are related to the results described below. Because implant treatment always involves surgical procedures, the oxidative stress resulting from inflammation often occurs around the implant. Excess amounts are known to cause delayed cell apoptosis, tissue healing, and implant placement failure. In other words, the suppression of ROS production is important for the successful implant treatment. As mentioned above, argon plasma treatment forms active oxygen on the surface of the material. In this study, ROS evaluation using bone marrow cells revealed that the argon plasma treatment on titanium surfaces suppressed the occurrence of oxidative stress on the material surface. Few reports show that the reduction of carbon on the material surface is caused by the suppression of ROS production [53,54,55].

The surfaces of all the implant materials were covered with a layer of protein upon implantation. The presence of protein layers regulates the response and behavioral cascade of the bone marrow cells [56]. Albumin is the most abundant plasma protein. In this experiment, the plasma-treated group adsorbed more albumin than the control group and showed the highest value in the argon plasma-treated group. In addition, the argon plasma-treated titanium surface enhances the adhesion of cells, such as osteoblasts and fibroblasts. Therefore, it was proposed that the changes in the surface energy of a material may promote tissue growth by increasing the adsorption of certain proteins compared with the materials with microscale characteristics. RBMCs on the surface of titanium metal, treated with argon plasma, exhibited higher ALP activity, calcium deposition, and bone formation-related gene expression, than RBMCs on the surface of the untreated control group. ALP activity is an indicator of early-stage bone differentiation, bone formation, and osteoblast activity [57]. There was a significant difference between the surface of the titanium implant treated with atmospheric pressure plasma and the surface of the untreated titanium implant, with respect to the expression level of the osteoblast-specific marker. In addition, the plasma-treated surface upregulated ALP, Bglap, BMP-2, and Runx2 in the RBMCs. Plasma treatment maintains the viability of the attached stromal cells and promotes their differentiation into osteoblasts.

In the in vivo evaluation of this study, the femur of a rat was used, and the implanted material was in direct contact with the sea surface bone, assuming an actual dental clinic. In a rat model, healing 8 weeks after transplantation is considered the final stage of wound healing because it allows bone marrow cells to contact the surface of the implanted implant material [58,59]. This is the key to osseointegration. The above-mentioned in vitro evaluation revealed that the surface of the pure titanium metal in the argon plasma treatment group showed the highest initial adhesion ability of bone marrow cells. In other words, it is clear that our in vivo analysis results correlate with the in vitro evaluation. In this study, the results of micro-CT and histopathological diagnosis showed an increase in BIC, BV/TV, and Tb.Th values, and a decrease in Tb.Sp around the argon-plasma-treated implants. Therefore, the surface of the implant material subjected to argon plasma surface control significantly increases the adsorption of protein due to the superhydrophilicity of the material surface, and the bone contact rate is increased by contacting the surface bone of the rat femur. It was revealed that there was a significant increase. However, a strong evidence has not been obtained yet, and it should be investigated in the future studies.

## 4. Materials and Methods

### 4.1. Sample Preparation

**Figure 13:**
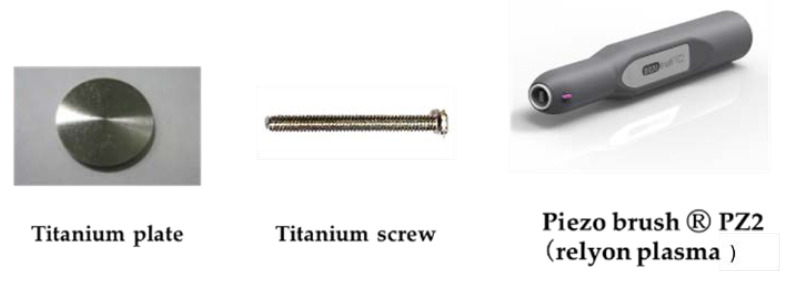
Titanium samples and titanium screw implants were used in this study for in vitro and in vivo analysis, respectively. The plasma treatments on the titanium surface were performed using a piezobrush^®^. PZ2.

This study was designed to evaluate the effect of argon-based atmospheric pressure plasma treatment on hard tissue formation on titanium surfaces. The experimental group was divided into three groups: argon-based atmospheric pressure plasma-treated group, atmospheric pressure plasma-treated group, and untreated group. Titanium samples (JIS Grade 2, 15 mm in diameter, and 1 mm-thick titanium, Daido Steel, Osaka, Japan) and titanium screw implants (1.2 mm in external diameter and 12 mm in length, Daido Steel) were used in this study for in vitro and in vivo analysis, respectively (Figure 13). The disks and screw implants were ultrasonically rinsed in acetone, ethanol, and distilled water for 10 min each, and air-dried. The plasma treatments on the titanium surface were performed using a piezobrush^®^ PZ2 (Relyon Plazma GmbH, Regensburg, Germany). The plasma treatment was performed using an active gas under atmospheric pressure; the low-temperature plasma treatment was conducted under irradiation at 0.2 MPa for 30 s at 10 mm, and the argon-based atmospheric pressure plasma treatment was performed under irradiation at 0.1 MPa for 30 s at 10 mm.

### 4.2. Characterization of Materials

The surfaces of the titanium materials were observed using a scanning electron microscope (S-4800; Hitachi, Tokyo, Japan) and a scanning probe microscope (SPM-9600, Shimadzu Corporation, Kyoto, Japan). The chemical composition of the surface of the various titanium materials were analyzed via XPS (Kratos Analytical Axis super-DLD Electron Spectrometer; Kratos Instruments, Manchester, UK) using a monochromatic AlKαX source. The contact angle measurements of the titanium surface were performed using a video contact angle measurement system (SImage Entry 6; Excimer Inc., Kanagawa, Japan).

### 4.3. Protein Adsorption

Bovine serum albumin, fraction V (Pierce Biotechnology) was used as a model protein. Three hundred microliters of protein solution (1 mg/mL protein in saline) was pipetted onto each specimen. After incubation for 1, 3, 6, and 24 h at 37 °C, non-adherent proteins were removed and mixed with bicinchoninic acid (Pierce Biotechnology) at 37 °C for 1 h. The amount of the removed albumin as well as the total amount of the albumin inoculated were quantified using a microplate reader at 562 nm. The rate of albumin adsorption was calculated as the percentage of the albumin adsorbed onto the specimens relative to the total amount.

### 4.4. Cell Culture

RBMCs were obtained from the femurs of 8-week-old Sprague–Dawley rats (SHIMIZU Laboratory Supplies Co., Kyoto, Japan). The method of extraction of RBMCs followed the procedures employed in our previous reports. The animal experiments were performed according to the ethical principles of the National Animal Care Guidelines and were approved by the Medical Ethics Committee of Osaka Dental University, Japan (approval no. 19-06001). RBMCs were removed from the respective flasks and seeded at a density of 4 × 10^4^ cells/well into the 24-well tissue culture plates (BD Biosciences, Franklin Lakes, NJ, USA) containing titanium disks of the three groups.

### 4.5. Cell Adhesion

RBMCs were seeded onto titanium surfaces of three groups at an initial density of 4 × 10^4^ cells/cm^2^ and allowed to adhere to the surface for 1, 3, 6, and 24 h. The number of RBMC adhesions was examined using the CellTiter-Blue^®^ Reagent (50 μL CellTiter-Blue^®^ Reagent diluted in 250 μL PBS). The analysis was performed according to the manufacturer’s instructions. According to the procedure established in our previous report, the cells were stained and observed after 6 h of culture.

### 4.6. qRT-PCR, Alkaline Phosphatase Activity, DNA Content, and Mineralization Determination

The expression of osteogenesis-related genes was assessed using a real-time TaqMan RT-PCR assay (Life Technologies, Carlsbad, CA, USA). The total RNA was extracted using the RNeasy Mini Kit (Qiagen, Venlo, The Netherlands), and 10-μL aliquots of each RNA sample were reverse transcribed into cDNA using a Prime Script RT Reagent kit (TaKaRa Bio, Shiga, Japan). We investigated the mRNA expression of ALP on day 7, runt-related transcription factor (Runx2) on day 3, bone morphogenetic protein 2 (Bmp-2) on day 14, and Bglap on day 21, as osteogenesis markers.

To evaluate the ALP activity after days 7 or 14 of incubation, the samples were washed with PBS, and the cells that adhered to the sample surface were dissolved in 300 μL of 0.2% Triton X-100. The ALP activity was evaluated using an alkaline phosphatase luminometric enzyme-linked immunosorbent assay (ELISA) kit (Sigma-Aldrich, Steinheim, Germany)) according to the manufacturer’s instructions. A PicoGreen dsDNA analysis kit (Invitrogen/Life Technologies) was used to evaluate DNA content. The amount of ALP was normalized to the amount of DNA in each cell lysate.

After days 21 or 28 of incubation, calcium deposition in the extracellular matrix was measured after dissolution with 10% formic acid. The calcium content was quantified and calculated using a Calcium E-test Kit (Wako Pure Chemical Industries Ltd., Tokyo, Japan), according to the manufacturer’s instructions.

### 4.7. Cell Intracellular ROS Level and Mitochondrial Membrane Potential Change Detection of RBMCs

Intracellular ROS levels were determined using the CellROX^®^ oxidative stress reagent (C10422, Thermo Fisher Life Technologies Ltd., Tokyo, Japan). The cells were washed with PBS three times and incubated in a medium containing 5 μM CellROX^®^ oxidative stress reagents for 30 min at 37 °C. rBM-MSCs were collected by trypsinization and transferred to a 96-well plate. ROS levels on rBM-MSCs of titanium disks of the three groups were stained and observed via confocal laser scanning microscopy (LSM700, Manufacturer, City, Zeiss, Germany).

### 4.8. Animal Model and Surgical Procedures

Male Sprague–Dawley rats (Shimizu Laboratory Supplies Co., Kyoto, Japan) were used in this study at 8 weeks of age. The rats were randomly divided into three groups, with eight rats in each group. The surgical procedures used in this study have been described previously. After general anesthesia and surgical cleaning, a 10-mm longitudinal incision was made along the medial side of the knee joint of the right hind leg. The patella and extensor were subsequently dislocated to expose the distal femur. A 1.2-mm hole was drilled into the intercondylar notch using a dental burr with sterilized saline irrigation. Screws were implanted into the prepared channels, the knee joint was restored, and the incision was sutured. Gentamicin (1 mg/kg) and buprenorphine (0.05 mg/kg) were injected for three days after surgery to prevent post-surgical infection and to decrease postoperative pain.

### 4.9. Morphological Analysis

After labeling, the rats were anesthetized and euthanized at 8 weeks, and the right femurs, including the implants, were placed in a saline solution immediately after dissection and scanned using an SMX-130CT microcomputed tomography (micro-CT) scanner (Shimadzu), operated at 70 kV and 118 mA. Three-dimensional reconstruction models were obtained using a morphometric software (TRI/3D-BON; Ratoc System Engineering, Tokyo, Japan). The region of interest was defined at 2 mm below the highest point of the growth plate, extending 500 μm around each implant. The bone volume fraction (BV/TV), mean trabecular number (Tb.N), and mean trabecular thickness (Tb.Th), were quantified to assess bone regeneration.

After the micro-CT scan, implanted femurs collected at 8 weeks were stained using the Villanueva method to evaluate bone generation. All histomorphometric and fluorescence characteristics of the sections were analyzed using a BZ-9000 digital cold illumination microscope (Keyence Co., Osaka, Japan) and a laser scanning microscope (Carl Zeiss, Oberkochen, Germany), respectively. The bone area, BIC, and the labeled bone area were assessed using ImageJ software with a 200× field around the implant.

### 4.10. Statistical Analyses

In vitro evaluation was performed four times each. In vivo evaluation used the femurs of eight rats in each group. The data were analyzed using SPSS software (version 19.0; SPSS, Chicago, IL, USA). The one-way analysis of variance, followed by a Student–Newman–Keuls post hoc test was conducted to determine the level of significance. When a significant difference was found, Bonferroni multiple comparison was used. Statistical significance was set at *p* < 0.05, and *p* < 0.01 was considered highly significant.

## 5. Conclusions

This study found that carbon on the surface of the material was removed, and the surface hydrophilicity of the pure titanium metal surface treated with argon plasma was improved. In addition, the formation of ROS and nitrogen compounds and the suppression of the development of oxidative stress on the surface of the material further improved its ability to induce early adhesion of rBM-MSCs and hard-tissue differentiation. This study also revealed the formation of new bones in the tissue surrounding the implant.
